# Small molecule compounds targeting the p53 pathway: are we finally making progress?

**DOI:** 10.1007/s10495-014-0990-3

**Published:** 2014-04-23

**Authors:** Xin Yu, Sumana Narayanan, Alexei Vazquez, Darren R. Carpizo

**Affiliations:** 1 Rutgers Cancer Institute of New Jersey, Robert Wood Johnson Medical School, Rutgers—The State University of New Jersey, 195 Little Albany Street, New Brunswick, NJ 08903 USA; 2 Department of Radiation Oncology, Robert Wood Johnson Medical School, Rutgers—The State University of New Jersey, 195 Little Albany Street, New Brunswick, NJ 08903 USA; 3 Department of Surgery, Robert Wood Johnson Medical School, Rutgers—The State University of New Jersey, 195 Little Albany Street, New Brunswick, NJ 08903 USA

**Keywords:** p53, Small molecule compound, Wild-type, Mutant, Reactivation

## Abstract

Loss of function of p53, either through mutations in the gene or through mutations to other members of the pathway that inactivate wild-type p53, remains a critically important aspect of human cancer development. As such, p53 remains the most commonly mutated gene in human cancer. For these reasons, pharmacologic activation of the p53 pathway has been a highly sought after, yet unachieved goal in developmental therapeutics. Recently progress has been made not only in the discovery of small molecules that target wild-type and mutant p53, but also in the initiation and completion of the first in-human clinical trials for several of these drugs. Here, we review the current literature of drugs that target wild-type and mutant p53 with a focus on small-molecule type compounds. We discuss common means of drug discovery and group them according to their common mechanisms of action. Lastly, we review the current status of the various drugs in the development process and identify newer areas of p53 tumor biology that may prove therapeutically useful.

## Introduction

Over the past several decades, anti-cancer drug development has witnessed a number of examples of targeted molecular agents succeed in the clinic. These include imatinib for bcr-abl chronic myelogenous leukemia (CML) and c-kit positive gastrointestinal stromal tumors (GIST), as well as gefitinib for EGFR mutant tumors [1–3]. While promising results with these drugs validate the concept of targeted therapy, the number of patients with these mutations is relatively small compared to the number of patients with mutations in *TP53*, *RAS*, and *MYC*. These are the most commonly mutated genes in human cancer, for which there are no effective targeted drugs available in the clinic today. Nonetheless, efforts to target these genes therapeutically remain an area of intense cancer research. With respect to p53, the field is making progress with several drugs now in phase I clinical trials and new lead compounds being developed.

**Fig. 1:**
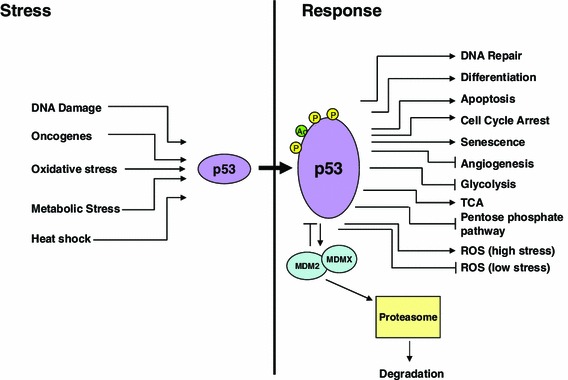
The p53 pathway. p53 is a critical responder to various modes of cellular stress substantiating its role as a key tumor suppressor in cancer biology. These modes serve to activate p53 both by stabilizing the protein (decreasing its MDM2 mediated proteasomal degradation) and enhancing its function as a transcription factor. The response to p53 encompasses a wide range of cellular processes that allow the cell to recover/repair the damage induced by the stress. The determination of which effector pathway it uses is dependent on several variables including the source of the stress, the cell type and surrounding microenvironment

Over 30 years of research on the p53 tumor suppressor has substantiated it as one of most critically important genes in human tumor biology [4]. p53 is a transcription factor whose primary function is to maintain cellular homeostasis in response to genotoxic stress signals by different means including upregulation of genes involved in cell cycle arrest, apoptosis, senescence and metabolism (Fig. 1) [5, 6]. Given this role, it is obvious why so many human tumors require the loss of function of p53 to progress to a fully malignant phenotype. While p53 exhibits some classical features of a tumor suppressor including loss of heterozygosity (LOH), it is distinguished by the frequency of missense mutations found in the gene. Indeed, the majority of mutations (>70 %) are single amino acid missense mutations that generate a defective and abundant protein. This latter fact is highly important to anti-cancer drug researchers as it allows p53 to be potentially targetable.

There are several aspects of the biology of mutant p53 that make it an attractive target for drug development. (1) The target (mutant p53) protein is found at high levels in cells. Wild-type p53 protein levels are tightly regulated by the E3 ubiquitin ligase (MDM2) which targets wild-type p53 for proteasomal degradation. As an E3 ubiquitin ligase, MDM2 can also degrade itself as a negative feedback [7]. In cancer cells with mutant p53, this regulation is lost in part, due to the inability of p53 to transcriptionally upregulate MDM2 [8, 9], as well as the binding of mutant p53 to the heat shock protein 90 complex (Hsp90) which prevents ubiquitination of mutant p53 [10]. (2) Lessons learned from murine models of cancer indicate that restoring the function of wild-type p53 in tumors is highly therapeutic and in some instances curative [11–13]. (3) There is a growing body of evidence that mutant p53 proteins exhibit a wide range of tumor biology that goes beyond their loss of wild-type p53 transcriptional function and collectively has been termed the mutant p53 gain of function (GOF) phenotype [14]. These proteins have been implicated in enhanced tumorigenesis, invasion and metastasis thus targeting them could reap further therapeutic rewards [15–18].

While the majority of tumors that have lost the function of p53 contain missense mutations, there are also a large proportion of tumors with wild-type p53 that have impaired p53 signaling due to dysregulated or mutated proteins in the pathway. Examples of this include increased degradation of p53 by increased MDM2 activity, which can result from overexpression of MDM2 in tumors (i.e. MDM2 amplifications) or loss of p19^ARF^ [19, 20]. Alternatively overexpression of the negative regulator MDMX can result in a similar effect (impaired p53 signaling) [21]. For this reason, considerable attention has been given to the investigation of small-molecules that can increase the activity of wild-type p53 through targeting MDM2 and/or MDMX. However, there are still other ways in which p53 signaling is dysregulated that can have an impact on how agents that target wild-type p53 perform. For example overexpression of the WIP-1 phosphatase (dephosphorylates and inactivates p53) through gain of function mutations has been reported as means of inactivating p53 in human tumors [22].

Here, we review the current research of p53 targeted drug development focusing on small molecule compounds. We also provide an overview of current strategies employed to identify compounds that activate wild-type p53 and restore wild-type function of mutant p53, as well as discuss some of their pitfalls and obstacles to clinical translation. While several other strategies have been employed to restore p53 function in tumors including the use of stapled peptides and gene therapeutic approaches (reviewed in [23, 24]), this review will focus on small molecules that target wild-type and mutant p53 (Table 1).

**Table 1:** Compounds and small molecules that target wild-type and mutant p53

Molecule/compound	Mechanism of action	Target	Stage of development
Activate wild-type p53			
Nutlins RG7112 (RO5045337)	Inhibits p53-MDM2 binding	MDM2	Phase I clinical trial (NCT01164033, NCT01143740, NCT00623870 and NCT00559533) [48]
Benzodiazepinediones (TDP665759)	Inhibits p53-MDM2 binding	MDM2	Preclinical [50]
Spiro-oxindoles (MI-219)	Inhibits p53-MDM2 binding	MDM2	Preclinical [13]
RITA	Inhibits p53 binding	p53 (WT and mut)	Preclinical [53]
JNJ-26854165 (Serdemetan)	Inhibits p53-MDM2 binding	MDM2	Phase I clinical trial (NCT00676910) [58, 60]
Tenovin 1 and 6	Inhibits SirT1 and SirT2 (protein deacetylators)	SirT1 and SirT2	Preclinical [117]
SJ-172550	Inhibits p53:MDM2/X binding	MDMX	Preclinial [69]
RO-2443/RO-5693	Inhibits p53:MDM2/X binding	MDMX	Preclinical [70]
XI-011	Repression of MDMX promoter	MDMX	Preclinical [71]
Re-activate mutant p53			
CP-31398	Interacts with DNA, reactive oxygen species	V173A, R175S, R249S, R273H	Preclinical [83]
PRIMA-1 (APR-246)	Covalently modifies cysteine residues, protein folding	R273H, R175H	Phase I clinical trial (NCT00900614) [87]
MIRA-1	Alkylation Cysteine and lysine residues	R175H, R248 W, R248Q, R273H, R282W	Preclinical [88]
PhiKan083	Slows thermal denaturation	Y220C	Preclinical [25]
NSC319726	Zinc chelation/Redox modulation	R175H	Preclinical [32]

## Strategies for p53 targeted drug discovery

The pharmacological inhibition or reactivation of transcription factors by small molecules is a challenging task. However, different strategies have been employed to uncover small molecules that reactivate mutant p53 proteins. These strategies can be divided into a few major categories based on the readout of the primary screen: structural stability, trans-activation of p53 targets, growth inhibition or synthetic lethality.

### Reactivation of structural stability

Approximately one-third of p53 mutations result in structurally destabilized proteins [25]. Structure based approaches aim to identify small molecules that stabilize protein structure. This approach applies x-ray crystal and NMR structures to identify pockets within the protein that can serve as templates for which compounds can be designed to potentially interact. Then in silico screens are performed to identify small molecules that bind to those pockets and potentially stabilize the protein structure.

Boeckler et al. [25] screened 2,066,906 compounds to identify compounds that bind to the p53-Y220C core domain crystal structure. The Y220C mutation creates a surface crevice destabilizing the p53 protein [26]. 80 candidate compounds were then tested on an in vitro screen to identify compounds that induce a chemical shift as detected by NMR spectroscopy. One compound (PhiKan083) was found to bind to the mutation-induced cleft in Y220C.

Based on the crystal structure of the p53 wild-type DNA binding domain, molecular dynamics simulations and genetic studies, Wassman et al. [27] identified the pocket between loop L1 and sheet S3 of the p53 core domain as a potential target for small molecules. Subsequently, they in silico screened 1,324 compounds from the NCI/DTP chemical repository for the ability to bind to that pocket. Using this approach they identified stictic acid (NSC87511) as a candidate p53 reactivating compound. In follow up validation studies they observed that stictic acid was able to induce p21 and PUMA in a dose and p53 dependent manner in Saos-2 p53-null cells transfected with R175H and G245S relative to untreated controls.

### Reactivation of p53 transcriptional activity

The most frequent mutations in p53 result in the change/loss of wild-type transcriptional activity. Interestingly mutant p53 retains transcriptional function through both direct and indirect mechanisms [28]. Nonetheless, reactivation of wild-type p53 transcriptional activity has been sought as a good indicator of the success of a chemical screen.

Wang et al. [29] tested approximately 2,000 compounds from the NCI/DTP chemical library for their ability to activate a p53-responsive promoter or cause cell death in HCT116 p53-null cells. Using this approach they identified several compounds with the ability to induce p53 target gene expression, cell cycle arrest and apoptosis in HCT116 p53-null cells. Interestingly, compounds with both p73 dependent and p73 independent activity were identified. The ability to reactivate p53 target expression was further validated in vivo using DLD1 (p53-null) tumor xenografts.

Kravchenko et al. [30] screened 46,260 compounds for their ability to activate p53-response promoters in the A431 cell line bearing a R273H mutant. Using this approach they identified a small-molecule (reactivation of transcriptional reporter activity, RETRA) that induced expression of the p53 homologue, p73 and its release from complex with mutant p53. Follow up studies showed that RETRA induced apoptosis in A431 (R273H) cells in a p73 dependent manner and significantly reduced tumor formation in A431 xenografts.

### Reactivation of p53-dependent growth inhibition

Wild-type p53 is activated by a variety of extrinsic and intrinsic stresses, inducing transcriptional programs that can lead to cell cycle arrest or apoptosis. When p53 is mutated p53-dependent growth inhibition is lost. Reactivation of p53-dependent growth inhibition has been also exploited for chemical screens.

Bykov et al. [31] screened compounds from the NCI/DTP library for their ability to inhibit growth in several human tumor cell lines carrying a tetracycline-regulated R273H or R175H and identified PRIMA-1, a compound that inhibited growth in a mutant p53 dependent manner. Follow up validation studies showed that PRIMA-1 restored proper folding of R175H, DNA binding, p53 target gene expression and induced apoptosis in the cells. In vivo PRIMA-1 selectively induced apoptosis and reduced tumor growth of Saos-2 p53-R273H relative to Saos-2 p53-null mouse xenografts.

We have conducted an in silico screen for compounds that preferentially inhibit the growth of p53 mutant cells relative to p53 wild-type cells [32]. Our screen was based on data from the NCI/DTP anticancer drug screen, reporting the IC50s for about 50,000 compounds against 60 tumor derived cell lines [33], and the reported p53 status of those cell lines [34]. A major difference compared with previous screens is the evaluation of heterogeneous panels of cell lines rather than engineered isogenic cell lines. This screen uncovered three thiosemicarbazones which preferentially inhibited p53 mutant cell lines relative to p53 wild-type cells. One of these compounds, NSC319726, showed allele specificity against the R175H carrying cells in vitro and in vivo mouse xenografts. This specificity is in part due to a refolding of R175H to a wild-type like conformation.

## Limitations of screening strategies

Screening methodologies aiming to identify compounds reactivating a p53 response have caveats and limitations. Screens based on the transcription of a p53 promoter may identify compounds that alter the DNA conformation by intercalating into DNA, resulting in DNA damage [35]. Screens based on a growth inhibition or other phenotypic assays may identify compounds that do not act directly through the action of the p53 molecule. Yet, these compounds may still be interesting from the point of view of synthetic lethality. Compounds aiding the refolding of structural mutants into a wild-type conformation may still not be active because they do not trigger the post-translational modifications that are necessary to obtain a p53 response. From the work reviewed above, it is evident that these methodologies are tailored for very specific alleles, including wild-type p53 and different point mutations. However, the sum of all the methodologies explored so far does not cover all p53 alleles. The investigation of strategies to identify compounds targeting cancer cells harboring p53 point mutants that are not classified as DNA binding or structural mutations is still missing. Furthermore, targeting p53 null cells requires a different approach altogether, which can be based on the identification of synthetic lethality (increased sensitivity to inhibition of another pathway in the absence of p53) or induction of a p53-like response by the activation of the p53 family member s p73 or p63 [29].

## Targeting wild-type p53

**Fig. 2:**
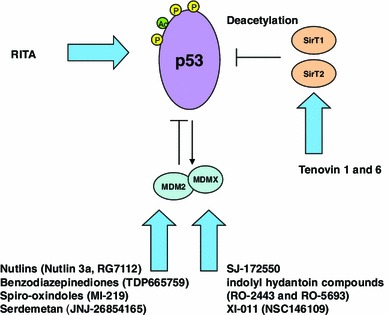
Mechanisms of action for drugs that target wild-type p53—a number of compounds now exist that serve to enhance the function of wild-type p53 by increasing the stability of p53 through various mechanisms: The largest group (nutlins, benzodiazepinediones and spiro-oxindoles) serve to increase the stability of p53 by targeting the MDM2:p53 interaction. This leads to decreased MDM2 mediated proteasomal degradation of p53. MDMX inhibitors block the MDMX-p53 interaction so as to activate wild-type p53. RITA also stabilizes wild-type p53 supposedly through binding p53 and inducing a conformational change that disrupts the p53:MDM2 binding, though this is controversial. Other compounds (tenovins) enhance the stability of p53 through inhibition of the sirtuins

There are two major mechanisms of action for compounds that increase the activity of wild-type p53 (Fig. 2). The first involves increasing wild-type p53 levels by interfering with the MDM2 mediated proteasomal degradation of p53. The other is through targeting enzymes that negatively regulate p53 through post-translational modifications. The p53 protein is under tight regulation by MDM2, an E3 ubiquitin ligase that ubiquitinates p53 and targets it for proteasomal degradation [36]. MDM2 itself is transcriptionally regulated by p53 which forms a negative feedback loop allowing for the inhibition of the p53 protein and subsequent decrease in MDM2 levels [37]. MDM2 is over-expressed via gene amplification in many human tumors, which effectively decreases wild-type p53 function [6]. Numerous studies have corroborated the notion that decreasing MDM2 levels (by various means biochemically or genetically) leads to an increase in p53 activity [38–41]. As a result considerable efforts have been made by drug researchers to develop compounds that interfere with the p53:MDM2 interaction leading to the supposition that inhibition of MDM2 may lead to re-activation of wild-type p53 in cancer cells [42].

Targeting the p53:MDM2 interaction has been greatly benefited by elucidating the x-ray crystal structure of the amino-terminal domain of MDM2 and a 15 amino acid peptide sequence of the transactivation domain of p53 [43]. This structure revealed that at the MDM2:p53 interface there is a well-defined hydrophobic pocket that contacts three residues on p53 (Phe19, Trp23 and Leu26) that could potentially be the target of a small molecule inhibitor [44]. The nutlins, benzodiazepinediones and spiro-oxindoles are three groups of small molecules that have been found through a variety of chemical screens to bind to MDM2 and prevent p53 binding.

### Nutlins

Nutlins are a group of cis-imidazoline small-molecule compounds identified from a large chemical screen and chosen for their potency and selectivity for inhibition of the MDM2:p53 interaction, with IC50 values from 100 to 300 nM [45]. Nutlin-3a, when used at micromolar concentrations, arrested proliferating cancer cells in the G1 and G2 cell cycle phases as well as induced apoptosis in wild-type p53 dependent manner, in a number of different cancer cell lines including colorectal, lung, breast, prostate, melanoma, osteosarcoma and renal cancer, all of which expressed wild-type p53 when studied by Tovar et al. [46]. Every cell line treated with Nutlin-3a demonstrated dose dependent p21 induction and cell cycle arrest [46]. For reasons pertaining to pharmacologic properties of Nutlin-3a, further lead optimization was required to generate a compound for clinical development. RG7112 is a potent and selective member of the nutlin family that has been optimized pharmacologically and can be given orally [47]. It has been shown in human xenograft models to inhibit wild-type p53 tumor growth in a dose dependent fashion and even exhibited regression in some instances. RG7112 (RO5045337) is currently in phase-I clinical trials (NCT01164033, NCT01143740, NCT00623870, and NCT00559533) in advanced solid tumors, hematologic malignancies and liposarcomas and was shown to be well tolerated and demonstrating some clinical activity [48].

### Benzodiazepinediones

The Benzodiazepinediones are benzodiazepine derivatives that disrupt the MDM2:p53 interaction in a similar manner to the nutlins by mimicking the action of the key amino acids involved in the binding of the p53 peptide to MDM2 [49]. The compounds that interacted with MDM2 were identified when screening of a library of 22,000 benzodiazepinediones. The compounds that bound to MDM2 were identified by their increased thermal stability using ThermoFluor microcalorimetry to monitor temperature dependent protein folding [50]. The benzodiazepinedione compound TDP665759 was found to bind to MDM2 with an IC50 of 704 nM and inhibit proliferation in cell lines expressing wild-type p53 [50].

### Spiro-oxindoles

The spiro-oxindoles are small molecule MDM2 inhibitors designed via computational modeling to activate wild-type p53 by mimicking the p53 amino acid side-chains which interact with the MDM2 binding pocket [49]. Ding et al. [44] determined that the indole ring of the Trp23 amino acid residue was vital for p53 to fit into the hydrophobic pocket of MDM2 and thus developed oxindole based small molecules that could mimic this interaction. An advantage of the spiro-oxindoles is that they have specificity against cancer cell lines versus normal epithelial cells [44]. One of these compounds, MI-219, has been shown to inhibit cancer cell proliferation and to inhibit tumor growth in xenograft models. It also has good tissue bioavailability in mice and is orally active [51, 52].

### Reactivation of p53 and induction of tumor cell apoptosis (RITA)

RITA is a compound identified via phenotypic screen using isogenic colon cancer cell lines HCT116 (wild-type p53 vs. null). Using a cell proliferation assay, compounds from a National Cancer Institute (NCI) database were tested to assess whether they suppressed the growth of the wild-type cell line [53]. Using fluorescence anisotropic experiments, it was shown that RITA directly binds p53 and causes a conformational change that prevents the binding between p53 and MDM2, effectively inducing apoptosis in cells with wild-type p53, but not mutant p53 or null. Moreover, RITA has been shown to inhibit p53-wild-type xenograft tumor growth when administered intra-peritoneally. However, the exact mechanism that RITA uses to activate wild-type p53 remains unclear. Recently heteronuclear single-quantum coherence (HSQC) NMR was used to determine if RITA bound p53 in vitro. ^15−^N-labeled MDM2 (118 amino acid N-terminal domain) and p53 (312 amino acid N-terminal domain and DNA binding domains) fragments were synthesized and NMR spectra produced. RITA could not displace MDM2 binding in these experiments leading to the conclusion that RITA does not bind the N-terminus of p53 [54]. An alternative mechanism for RITA mediated p53 activation in multiple myeloma cells was recently described in which RITA transcriptionally induced p53 by activating JNK signaling which as its downstream effects leads to increased c-jun binding to the AP-1 binding sites in the p53 promoter. In these experiments inhibiting JNK signaling through an si-RNA to JNK inhibited RITA mediated p53 activation [55].

RITA has since also been found to suppress growth in a number of different cancers with mutant p53 such as colon, lung and breast carcinoma as well as Burkitt lymphoma with mutations at residues—273, 175 and 248, 280, 213, 234, 283, 254 and 125 [56]. RITA was shown to induce apoptosis in these mutant cell lines as well as transcriptionally activate p53 targets *p21*, *BAX*, *Noxa* and *PUMA* [56]. It is not clear how RITA can activate both wild-type and mutant p53. Nonetheless, RITA has been studied in combination with other drugs such as cisplatin where RITA enhanced cisplatin cytotoxicity through upregulation of p53 downstream apoptotic targets in head and neck cancer cells [57].

### JNJ-26854165 (Serdemetan)

JNJ-26854165 (Serdemetan) is a p53 activating tryptamine derivative that was initially thought to activate wild-type p53 by functioning as a E3 ubiquitin ligase inhibitor [7]. Kojima et al. [58] found that Serdemetan induced p53 dependent apoptosis and transcriptional activation of *p21* and *Noxa* in a number of leukemia cell lines. It was also found to induce early apoptosis (48 h) in cells with wild-type p53 status and delayed apoptosis (72–96 h) in mutant p53 cell lines [58]. Other pre-clinical studies have also found activity in both wild-type and mutant p53 tumors indicating that the mechanism involves both p53 dependent and independent functions [59]. Chargari et al. [60] found that Serdemetan significantly enhanced radiation induced growth delay in wild-type (H460 cell line) xenograft tumors as well as demonstrating G2/M cell cycle arrest in H460 and A549 cell lines.

Serdemetan was tested in a Phase I clinical trial in 5 study centers in Belgium and Spain in patients with advanced refractory solid malignancies (mostly colorectal cancers, sarcomas and melanomas). Serdemetan was found to be rapidly absorbed orally and maximum tumor reduction was seen in patients receiving above 150 mg/day, the threshold for induction of p53 in skin biopsies [61]. This compound is no longer in clinical development.

### MDMX inhibitors

MDMX is a partner protein to MDM2 that is structurally similar at the N-terminal domain where both proteins bind p53 [62]. While MDMX has no intrinsic E3 ubiquitin ligase activity, it does dimerize with MDM2 and MDM2/MDMX heterodimers not only enhance ligase activity but also are responsible for the polyubiquitination of p53 whereas MDM2 alone monoubiquitinates p53 [63, 64]. Genetic experiments in mice have demonstrated the importance of these MDM2/MDMX heterodimers in the negative regulation of p53 [65]. Owing to differences in the p53 binding sites between MDM2 and MDMX, MDM2 antagonists like Nutlin-3 have low affinity for MDMX and thus their ability to maximally inhibit p53 is diminished particularly in tumors where MDMX is over-expressed [66, 67]. This understanding has lead to the search for small molecules that inhibit MDMX as a means of activating p53 more robustly. Proof of this concept was recently demonstrated where a 12-mer peptide was identified that inhibited both the MDM2:p53 and MDMX:p53 interactions [68]. In cell lines over-expressing MDMX, this peptide demonstrated superior cell growth inhibition over Nutlin-3a. Since then several small molecules have been reported to activate wild-type p53 through targeting MDMX.

The first small molecule reported was SJ-172550, which was identified through a peptide-based high throughput screen and validated to kill cells over-expressing MDMX by reversibly binding MDMX [69]. These effects were found to be additive when SJ-172550 was administered in vitro with Nutlin-3a. Most recently, a series of indolyl hydantoin compounds RO-2443 and RO-5693 were also described as potent inhibitors of MDMX by binding to the p53 pocket of MDMX and inducing protein dimerization. RO-5693 activated wild-type p53 in a non-genotoxic fashion and was able to overcome the resistance of MDMX over-expressing cancer cells to Nutlin-3a [70]. Lastly another compound XI-011 (NSC146109) was reported to activate wild-type p53 in breast cancer cells by a mechanism that involved inhibition of MDMX through transcriptional repression of the MDMX promoter [71]. These compounds are all still very early in the development process but certainly validate the concept that MDMX blockade can overcome the limitations of MDM2 antagonists particularly in MDMX over-expressing tumors.

### Tenovin 1 and Tenovin 6

Tenovins belong to the group of compounds that activate wild-type p53 indirectly through targeting enzymes involved in negative regulation of p53. These are Sirtuins (such as SirT1 and Sir T2), a family of protein deacetylating enzymes. SirT1 has been shown to destabilize p53 by deacetylating one of its carboxy-terminal lysines (Lys382) which may lead to ubiquitination and proteasomal degradation [23]. Using a cell based screen, Tenovin 1 was identified to inhibit sirtuins activity [72]. A secondary compound Tenovin-6 is seven times more water soluble and is more cytotoxic. Tenovin 6 has also been shown to decrease tumor growth in vivo [23].

## Obstacles to clinical translation

There are several considerations that have still yet been resolved concerning the application of small molecule compounds that activate wild-type p53. One needs to consider the effect of activating wild-type p53 in normal non-cancerous tissues which could theoretically be toxic. Previous experiments of whole body radiation to mice as a means of activating p53 confirmed that the response to radiation is tissue specific with proliferative tissues being affected most significantly [73], thus bone marrow and intestinal toxicities are of concern with these drugs. The effect of activating p53 in normal tissues has been studied using mice carrying a hypomorphic allele of MDM2. These mice displayed a phenotype that was characterized by increased apoptosis in lymphocytes and epithelial cells that was p53 dependent [74]. This allele did not affect their development or lifespan but did affect their size. Thus it remains to be seen if these toxicities will also be found in patients as phase 1 testing of MDM2 antagonists is currently underway. It may be possible to mitigate these toxicities by administering p53 modulating drugs in combination with other therapies which would allow the administration of a lower dose of the p53 modulating agent [75, 76]. Another potential obstacle to the translation of activators of wild-type p53 is the acquisition of mutations in p53 during treatment. While it is known that genotoxic stress to cells with ionizing radiation or cytotoxic chemotherapy can induce mutations in p53, it was thought that this would not be an issue with MDM2 antagonists. Maki group recently demonstrated that treating wild-type p53 cancer cells chronically with Nutlin-3a could lead to resistant clones that acquired p53 mutations [77]. Nearly one-fifth of breast, colon and lung cancers over-express MDMX which is also a potential obstacle to the development of MDM2 inhibitors such as Nutlin-3a [21].

It is also possible that activation of p53 in tumor cells could lead to a senescence phenotype that might be problematic in the long term. Several laboratories have reported that the primary response in human tumor cells with wild-type p53 to genotoxic chemotherapeutic agents is not apoptosis but a form of stress induced premature senescence (SIPS) [78]. This could be problematic as these cells could escape this senescence and re-enter the cell cycle. The mechanisms that govern an apoptotic versus senescence p53 mediated phenotypes need to be further explored as these will impact the response to p53 targeted agents. Evidence that tissue specificity plays a role was reported when p53 expression was restored in a genetically engineered mouse model of Li-Fraumeni syndrome. Restoration of p53 function lead to apoptosis in lymphomas and senescence in sarcomas [12]. Lastly, a barrier to successful clinical translation for many targeted molecular agents is identifying additional drugs that can be used in combination to enhance efficacy. With respect to MDM2 inhibitors clues to which drugs to combine have revealed several candidates including cyclin dependent kinase inhibitors as well as adenoviral p53 mediated gene therapy [76, 79].

## Restoration of wild-type function of mutant p53

Eighty-one percent of the mutations in human tumors which have lost wild-type p53 function are missense mutations [80]. These mutations have been extensively characterized by the International Agency on Cancer Research (IARC) [81]. The vast majority (95 %) of these mutations occur within the DNA binding domain of p53 with six (hotspot) mutations occurring with a particularly high frequency [81]. These point mutations of p53 can be classified as either DNA contact or structural/conformational mutations. Contact mutations (such as those occurring at amino acids R273H and R248Q) have very little effect on the ability of the p53 protein to fold and therefore are very similar structurally to wild-type p53. On the other hand, structural mutations (such as R175H and R249S) have a significant effect on protein folding causing destabilization of the protein structure [37, 82]. Moreover, some point mutations are identified. These distinctions are important as we gain a greater appreciation for the phenotypic differences that these proteins impart to tumor cells as well as the fact that newer p53 mutant reactivators have exhibited allele specific effects.

### CP-31398

CP-31398 is a styrylquinazoline synthetic small molecule discovered in an in vitro assay by Pfizer that screened for molecules that protected the p53 core domain from denaturation upon application of heat [83, 84]. CP-31398 has been shown to increase expression of wild-type p53 targets, such as p21, when interacting with different p53 mutants such as 173 or 249 mutants in an Saos-2 osteosarcoma cell line [83]. In immunoblot experiments using a p53 null lung cancer cell transfected with mutant p53 (173 mutation), low micromolar concentrations of CP-31398 were found to increase PAB1620 (wild-type antibody) positive cells by five-fold after treatment for 6 h [83]. In mouse xenografts, twice daily injections of CP-31398 was found to completely inhibit growth of the colon tumor (241 mutation) and decreased tumor growth of melanoma cell line (249 mutation) by 75 % [83].

This compound was found to be initially promising; however Rippin et al. [85] determined by florescence experiments that CP-31398 intercalated into free DNA and remained bound when DNA complexed with p53, but had no detectable binding to the wild-type or R249S p53 core domain with concentrations up to 3 mM and concluded that this drug suppressed tumor cell growth in a p53 independent manner, likely due to its ability to intercalate into DNA [85].

### p53 reactivation and induction of massive apoptosis (PRIMA-1)

PRIMA-1 and its metabolite APR-246, have advanced the furthest in the drug development process amongst the drugs that target mutant p53. PRIMA-1 was identified by a chemical screen of an NCI chemical library that inhibited the growth of an osteosarcoma cell line (Saos-2) that carried a tetracycline-regulated mutant p53 (R273H) but had minor growth rate inhibition in tumor lines absent of mutant p53 (wild-type and null) [31].

Immunofluorescence experiments using conformation specific antibodies PAB1620 (recognizing wild-type conformation) and PAB240 (recognizing mutant conformation) showed an increase in wild-type conformation and a decrease in mutant conformation after treatment, indicating that PRIMA-1 induced a “wild-type” like conformation change to the p53-R175H mutant. This group also determined that PRIMA-1 could restore DNA binding in electromobility shift assays to a wide array of mutants that included both DNA contact (R248, R273) and conformational mutants (R175H) [31].

In a follow-up study by this group, Lambert et al. [86] reported on the mechanism of action of PRIMA-1. They determined that one of the decomposition products of PRIMA-1/PRIMA-1^MET^ has a chemically active double bond to covalently react with thiol groups in mutant p53. This was demonstrated with the addition of NAC that inhibited PRIMA-1 dependent apoptosis and growth suppression. Furthermore, it was suggested that PRIMA-1^MET^ induced oxidation in mutant p53 cells may contribute to its effects.

The first in-human clinical trial of a drug that targets mutant p53 was reported using APR-246 in 22 patients with hematologic malignancies and hormone refractory prostate cancer examining maximum tolerated dose (MTD), safety and pharmacokinetics [87]. This study used a standard dose-escalation scheme in which 3 patients were treated with each incremental dose using doses of 2, 3, 10, 30, 60 and 90 mg/kg. If no dose limiting toxicity (DLT) was observed then patients were treated with the next higher dose. If there was a DLT in one of the three patients, then the next group would be treated with the same dose. Overall there were 12 adverse events that were judged to be related to the study drug. The most common adverse events were fatigue, confusion, muscle aches, and sensory disturbances. These typically occurred during or near the end of the infusion and all were reversible with no bone marrow toxicity seen.

Pharmacokinetic studies demonstrated a plasma half-life of 4–5 h. Pharmacodynamic studies were performed on a limited number of patients with hematologic malignancies as they could sample circulating tumor cells before and after treatment. They performed cell-cycle, apoptosis and gene expression measurements for p53 downstream target genes on the circulating tumor cells. Three of twelve analyzed samples had p53 gene mutations with the remaining samples having wild-type status. The increase in p53 activity did not appear to correlate with p53 mutational status and further assessment of APR-246 needs to be made to assess its ability as a mutant reactivator [87].

### Mutant p53-dependent induction of rapid apoptosis (MIRA-1)

MIRA-1 is a maleimide derived molecule identified along with PRIMA-1 in a cellular screen. It appears to reactivate and restore apoptotic activity to mutant p53 (at residues R175H and R273H) by increasing DNA fragmentation and inducing caspase activity [82, 88]. Bykov et al. [88] demonstrated that MIRA-1 preserved the native conformation of both wild-type and mutant p53 (R175H and R248W) upon heating and also preserved sequence-specific DNA binding (R175H, R282W, R248Q, R248W/C176Y). MIRA-1 and its analog MIRA-3 were able to induce activation of target genes *p21*, *MDM2* and *PUMA* which was mutant p53 dependent [88].

### PhiKan083

PhiKan083 is a carbazole derivative that binds to a surface cavity created by the conformational mutation Y220C which highly destabilizes the p53 protein by 4 kcal/mol [25]. PhiKan083 was discovered via an in silico screen of the ZINC database based on its crystal structure and NMR spectroscopy and was shown to bind to the target surface crevice and raise the apparent melting temperature of the Y220C mutant by almost 2 °C, slowing its rate of thermal denaturation and increasing its half-life from 3.8 to 15.7 min [25, 89]. Most recently, PK7088 was found to change the Y220C conformation mutation and reactivate this mutant [90].

### Thiosemicarbazones

Several members of the thiosemicarbazone family were identified by an in silico screen of the NCI60 anti-cancer drug screen (NSC319725, NSC319726, NSC328784) as compounds that had preferential activity in mutant p53 cells lines while displaying relatively little toxicity in cell lines containing wild-type p53. Two of these compounds (NSC319725 and NSC319726) were validated and displayed increased sensitivity in cell lines expressing the p53-R175H mutant. Further study of NSC319726 (726) indicated that this compound induced apoptosis in R175H cells by restoring wild-type structure and function this mutant at doses that were completely nontoxic to normal human fibroblasts. Interestingly, this compound did not reactivate the contact mutants R248 or R273, thus displaying allele-specific p53 mutant reactivation. 726 inhibited xenograft tumor growth in mice that was R175H mutant dependent at relatively small doses of the drug (1 and 0.1 mg/kg) [32].

The mechanism of 726’s apparent R175H specific mutant p53 reactivational effects is currently unknown. There are some initial clues to the mechanism as it was found that the compound depends upon its zinc chelating and reactive oxygen species (ROS) inducing properties [32]. Supplemental zinc added to the media of R175H cells enhances the apoptotic effect of 726. Given that the R175H mutant is known as a “zinc-binding” mutant because this mutation impairs the protein’s ability to bind zinc, it is hypothesized that this compound may act as a zinc metallochaperone by donating zinc to allow this mutant to refold properly. Other groups have even demonstrated that supplemental zinc alone can induce such a conformational change in p53-R175H mutant cells [91]. Nonetheless, if 726 indeed functions as a zinc metallochaperone, it may also reactivate other mutants with impaired zinc binding.

Thiosemicarbazones have shown to induce ROS changes in cells by the creation of hydroxyl radicals through Fenton Chemistry [92]. Consistent with this, 726 was found to induce ROS levels in R175H mutant cells by measurements of the natural cellular dextoxificant, glutathione. Indeed these ROS changes induced by 726 are important to its mechanism as treatment with the detoxifying drug N-acetyl cysteine (NAC) abrograted much of the apoptotic activity of 726 in R175H mutant cells [32]. The exact role that these ROS changes play in the mechanism of 726 is currently unknown.

### Heat shock protein 90 inhibitors

It is well known that mutant p53 protein levels are high in cancer cells. This had been previously attributed to a loss p53 mediated transactivation of MDM2 [8, 9]. Recently, it has been demonstrated using p53 knock-in missense mutant mice that mutant p53 is degraded in an MDM2 mediated fashion in non-tumor tissues, and in a subset of tumor tissues indicating that other mechanisms are involved in stabilizing mutant p53 [93]. Once such mechanism that has been elucidated is the binding of mutant p53 to heat shock protein 90 (Hsp90) which serves to protect mutant p53 from ubiquitination. Knock-down of *HSP90* or pharmacologic inhibition with 17-allylamino-17-demethoxygeldanamycin (17-AAG) resulted in a release of Hsp90 from mutant p53 bound to MDM2 allowing ubiquitination and degradation [10]. Thus HSP90 inhibition theoretically might help reverse the mutant p53 gain-of-function phenotype (GOF) attributed to mutant p53.

## p53 and metabolism

Another emerging and exciting area of p53 research that has important implications for wild-type and mutant p53 targeted drug development is the role that p53 plays in the regulation of metabolism. It is now well known that the metabolism of cancer cells is altered in ways that provide a selective advantage for tumor progression and tumor maintenance [94, 95]. This includes alterations in glucose metabolism that favor anaerobic glycolysis (otherwise known as the Warburg effect), decreased mitochondrial respiration, and increased glutaminolysis to assist in both the replenishment of non-essential amino acids as well as in providing a carbon source for the synthesis of macromolecules (anaplerosis) [96]. This metabolic reprogramming is necessary for cancer cells to overcome both internal and external sources stress that would arrest or induce apoptosis in non-transformed cells. This metabolic transformation not only comes about as a response of the cell to changes in the extracellular environment (i.e. hypoxia), but also as a direct result of oncogene activation and/or loss of tumor suppressor function such as p53 [95].

How p53 functions as a tumor suppressor has largely been attributed to its role in mechanisms of cell cycle arrest, apoptosis and senescence. However, this concept has been recently challenged by several studies that taken collectively argue that p53’s tumor suppressive properties are likely mediated by other functions of p53. For example, mice lacking either PUMA, NOXA or p21 which are downstream of p53 in mediating both apoptosis, and cell cycle arrest fail to develop spontaneous tumors [97, 98]. Gu et al. recently generated p53 mutant mice with three lysine to arginine mutations at three positions in the DNA binding domain important for regulation of p53 by post-translational acetylation. These three mutations abated p53’s ability to regulate cell cycle arrest, apoptosis or senescence. Intriguingly these mice did not succumb to early onset tumor formation as did p53 null mice indicating that tumor suppression must be mediated by other p53 functions. When they interrogated the metabolic functions of this mutant, they found that this mutant could still regulate metabolic target genes such as glutaminase-2 and 3, and *TP53*-induced glycolysis and apoptosis regulator (TIGAR) as well as genes involved in the regulation of reactive oxygen species indicating that metabolic regulation may be one function of p53 that is involved in tumor suppression [99]. Exactly how p53 exerts its tumor suppression through its regulation of metabolism is not yet clear, but some emerging areas of research in p53 and metabolism point to several directions that potentially could impact developmental therapeutics.

One area focuses on the role that p53 plays in suppressing geroconversion [100]. Geroconversion is named for its relationship to the senescent or aging phenotype and is defined as the conversion from a reversible state of senescence (quiescence) to a state of irreversible senescence. This phenotype is thought to be pro-tumorigenic as these cells are hypertrophic, hypersecretory, hypermetabolic and hyper-inflammatory [101]. By suppressing geroconversion, p53 promotes quiescence which is characterized by a state of low protein synthesis, and metabolism. Signaling through the phosphoinositide 3-kinase (PI3K)/mammalian target of rapamycin (mTOR) pathway is thought to promote geroconversion and highlights the general reciprocal roles that the p53 and the mTOR signaling pathways play in the regulation of cell growth. TP53 negatively regulates the mTOR pathway through a number of means [102–104]. For example, it activates AMP-activated protein kinase (AMPK) by upregulating the sestrins in addition to upregulating a number of genes that negatively regulate the mTORC1 complex [105]. The PI3K/mTOR pathway is activated in large number of cancers which provides a rationale for the combination of a p53 modulating drug and an mTOR inhibitor [78, 106, 107].

Another area is p53’s regulation of the cellular processes that bioenergetics and macromolecular synthesis. It is here that p53 acts in a number of locations to influence these reactions through regulation of transcriptional targets. In general p53 functions to slow down glycolysis and speed up oxidative phosphorylation in essence opposing the Warburg effect [108, 109]. This is accomplished in part through activation of targets such as TIGAR (inhibits glycolysis through decreasing levels of fructose 2, 6-bisphosphate) and synthesis of cytochrome c oxidase (SCO2) (increases oxidative phosphorylation through SCO2 protein) [110]. By decreasing glycolysis, TIGAR allows glucose intermediates to be shunted through the Pentose Phosphate Pathway (PPP), which is essential for anti-oxidant function through the production of NADPH and reduced glutathione. While it is tempting to speculate that p53’s tumor suppressive function may be in part mediated through TIGAR, recent studies of the role of TIGAR in tumor formation have not supported this. Recently the function of TIGAR was studied by genetic deletion in a mouse model of intestinal adenoma formation [111]. In this model the absence of TIGAR leads to a decrease in tumor formation which may indicate that suppression of ROS may be necessary during early tumorigenesis. This illustrates the need for further research in understanding how p53’s role in metabolic reprogramming functions in tumor suppression.

Nonetheless, through our current understanding of the role of p53 in metabolic reprogramming, we can now begin to appreciate how this may exploited therapeutically. For example, the Pentose Phosphate Pathway (PPP) which among other things is a major mechanism for the cell to provide a renewed source of NADPH that is essential for regulating the redox state of cellular glutathione (GSH) and its oxidized form (GSSG) [112]. Without proper GSH levels, cells are vulnerable to an ROS mediated cell death. An example of this was illustrated recently in an elegant set of experiments in which the metabolic adaptations to serine starvation were studied in cancer cells with and without functional p53 [113]. Serine starvation of cancer cells caused them to shunt glucose from the glycolytic pathway towards the serine the biosynthesis pathway. This leads to an increase in flux through the tricarboxylic acid (TCA) cycle and hence an increased production of ROS. Importantly, p53 wild-type cells responded to this by maintaining production of cellular GSH synthesis while p53 deficient cells displayed reduced GSH. This critical difference caused the p53 deficient cells to undergo an ROS mediated cell death. This could represent an “Achilles heel” for cancer cells that lose p53 function by using drugs that mimic serine starvation, inhibit the PPP, or perhaps shunt the flux of glucose through the TCA cycle to take advantage of similar mechanisms to produce an anti-cancer effect. This concept has also been illustrated with the drug Metformin. Metformin activates AMPK which in turn can activate p53 (AMPK phosphorylates p53 on serine-15). In cells with functional p53, this results in a number of metabolic changes in the cell that among other things, induce autophagy. When tumor cells lack p53, this induction of autophagy does not occur and cells succumb to apoptosis [114].

Another role for p53 in cancer metabolism that might be relevant to drug development is the control of oxidative stress. Here the role for p53 is dual and seemingly opposing which seems to be related to the levels of p53. In situations of normal cellular function, basal levels of p53 control the transcription of several anti-oxidant genes (sestrin 1 (SESN1), sestrin 2 (SESN2), glutathione peroxidase 1 (GPX1)), which function to regulate the ROS that is produced by normal cellular respiration. Recently, the enzyme glutaminase-2 was found to be a p53 transcriptional target that serves to lower redox levels through the generation of GSH from glutamine [115]. In situations of significant stress, p53 can increase ROS levels through the transcription of a number of pro-oxidant genes such as *PUMA*, *NOXA*, and *NQO1* and carry out an apoptotic program (reviewed in [112]). It is this latter finding that may be relevant to mutant p53 targeted drug development because in mutant p53 cancer cells, the levels of the mutant protein are high. This may help trigger a pro-oxidant function in p53 upon application of a mutant p53 reactivating drug. This was demonstrated for the p53 reactivating thiosemicarbazone NSC319726 [32].

## Conclusion

Over three decades of research on p53 has substantiated it as one of the most critical cancer genes in human tumor biology uncovering an enormous potential for therapeutic activity. Yet we still today have not translated these findings into any approved p53 targeted agents. This has led some to question whether p53 targeted therapies represent an “empty promise” [116]. At this time, one cannot make this conclusion as there is clearly evidence of progress in the field. We now have agents that target wild-type p53 and mutant p53 in human clinical trials for the first time. It is important to be reminded that the drugs that are being tested in these clinical trials were based off of lead compounds that were discovered in the last 10 years [31, 45]. Moreover, new compounds have been identified for pre-clinical development as well as novel strategies for drug discovery. Some of these compounds employ mechanisms of action that contain clues to restore the function of wild-type p53 in additional p53 miss-sense mutants and thus require more investigation. Further study of combination therapies (e.g., involving conventional cytotoxic agents, or targeted agents such as mTOR inhibitors, administered in combination with p53 modulators) is also needed as these may overcome issues pertaining to toxicity and efficacy of p53 modulator monotherapy. Certainly, newer areas of p53 research such as stem cell biology, cancer metabolism and p53 mediated micro-RNA regulation will no doubt impact the field of p53 targeted therapy. In an era of personalized medicine, it will likely be important to design innovative clinical trials that are “proof of concept” type studies that enroll patients based on their p53 mutational status to continue to advance the field.
